# Measuring the health systems impact of disease control programmes: a critical reflection on the WHO building blocks framework

**DOI:** 10.1186/1471-2458-14-278

**Published:** 2014-03-25

**Authors:** Sandra Mounier-Jack, Ulla K Griffiths, Svea Closser, Helen Burchett, Bruno Marchal

**Affiliations:** 1 Department of Global Health and Development, London School of Hygiene and Tropical Medicine, 15-17 Tavistock Place, London WC1H 9SH, UK; 2 Department of Sociology and Anthropology, Middlebury College, Middlebury, VT, USA; 3 Department of Public Health, Institute of Tropical Medicine, Antwerp, Belgium

**Keywords:** Health systems, Framework, Methods

## Abstract

**Background:**

The WHO health systems Building Blocks framework has become ubiquitous in health systems research. However, it was not developed as a research instrument, but rather to facilitate investments of resources in health systems. In this paper, we reflect on the advantages and limitations of using the framework in applied research, as experienced in three empirical vaccine studies we have undertaken.

**Discussion:**

We argue that while the Building Blocks framework is valuable because of its simplicity and ability to provide a common language for researchers, it is not suitable for analysing dynamic, complex and inter-linked systems impacts. In our three studies, we found that the mechanical segmentation of effects by the WHO building blocks, without recognition of their interactions, hindered the understanding of impacts on systems as a whole. Other important limitations were the artificial equal weight given to each building block and the challenge in capturing longer term effects and opportunity costs. Another criticism is not of the framework per se, but rather how it is typically used, with a focus on the six building blocks to the neglect of the dynamic process and outcome aspects of health systems.

We believe the framework would be improved by making three amendments: integrating the missing “demand” component; incorporating an overarching, holistic health systems viewpoint and including scope for interactions between components. If researchers choose to use the Building Blocks framework, we recommend that it be adapted to the specific study question and context, with formative research and piloting conducted in order to inform this adaptation.

**Summary:**

As with frameworks in general, the WHO Building Blocks framework is valuable because it creates a common language and shared understanding. However, for applied research, it falls short of what is needed to holistically evaluate the impact of specific interventions on health systems. We propose that if researchers use the framework, it should be adapted and made context-specific.

## Background

### Introduction

**Figure 1:**
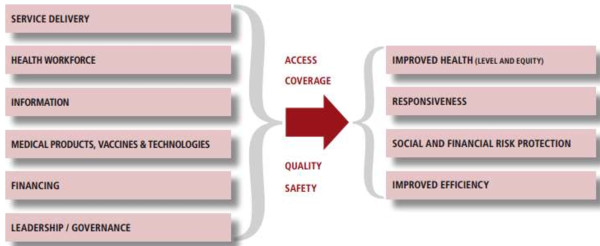
**The WHO health system building blocks framework.** Source: WHO 2007 [1].

In 2007, the WHO published a health systems Building Blocks framework with the aim of promoting a common understanding of what a health system is and what constitutes health systems strengthening [1]. In the framework, a health system is conceptualized as consisting of six building blocks: (i) service delivery; (ii) health workforce; (iii) information; (iv) medical products, vaccines and technologies; (v) financing; and (vi) leadership and governance, as well as process elements (access, coverage, quality and safety) and outcomes (improved health and health equity, responsiveness, social and financial risk protection and improved efficiency) (Figure 1) [1].

The WHO model is not the only model of health systems: Hoffman and colleagues identified 41 health systems frameworks developed since 1972 [2]. Nineteen of these encompassed the whole health system, while the remainder focused either on particular parts of the system or on how other societal systems interact with the health system. However, since its development the WHO Building Blocks framework has been widely used in health systems research and has arguably become the framework most often used to describe a health system in international forums [2].

Over the past few years, a lot of attention has been paid to health systems strengthening. Global health initiatives have developed specific funding instruments to foster such strengthening, for example the Global Alliance for Vaccines and Immunisation (GAVI)’s Health Systems Strengthening funding window [3]. There has been a growing recognition that the effects of new drugs and technologies, or other disease control strategies, may only have a limited effect if introduced into a weak health system. There have also been attempts by global agencies and researchers to evaluate the impact of vertical programmes on health systems, either to demonstrate positive impact or to identify potential negative effects [4-6]. In some cases, this has led to the development of specific guidelines and recommendations [7-9].

We have used the Building Blocks framework in three studies to evaluate the impact of disease control programmes on health systems: (i) measles eradication activities [9,10], (ii) new vaccine introductions [11] and (iii) polio eradication activities [12]. In the published literature, a number of other studies have used the Building Blocks framework for similar analysis, primarily in the field of HIV/AIDS and malaria [13-16].

Based on our experiences, the objective of this paper is to reflect critically on the usefulness and limitations of using the Building Blocks framework in applied research. We first present how the framework was applied in the three studies, and then discuss the utility and limits of the approach.

## Application of the building blocks framework in field studies

All three studies used a mixed method approach and used the Building Blocks as a conceptual framework. The measles study was primarily based on qualitative data, with most information collected through interviews and observations. The polio study used document review, interviews, and participant observation for qualitative data, and health utilisation data as well as national coverage data for quantitative analysis. The new vaccine study used semi-structured interviews, questionnaires and routine health service utilisation data. See Table 1 for more details.

**Table 1:** Study characteristics

**Programme**	**Evaluation methods used**	**Countries**
**Measles eradication**[10,17]	Semi-structured interviews with key informants (national, regional, district and facility levels)	Bangladesh, Brazil, Cameroon, Ethiopia, Tajikistan, Viet Nam
	Reviews of secondary documents	
**New vaccine introduction**	Semi-structured interviews with key informants (national, regional and district levels)	Cameroon Ethiopia Guatemala Kenya Rwanda, Mali
*PCV*	Questionnaire with health facility staff	
*Rotavirus*	Routine health service use data	
*HPV*		
*Meningitis A*[11]		
**Polio eradication**[12]	Semi-structured interviews with key informants (national, district and community levels)	Nepal, India, Pakistan, Nigeria, Ethiopia, Rwanda, Angola
	Participant observation in polio campaigns, surveillance, and routine health post activities	
	Reviews of documents	
	Routine health service use data	
	DPT3 coverage data from DHS and IHME	
	Attended births and antenatal care coverage data from DHS	

PCV = pneumococcal conjugate vaccine; rotavirus = rotavirus vaccine; HPV = human papillomavirus vaccine; Meningitis A = Meningitis A vaccine.

For all three studies, modified versions of the Building Blocks framework were used to develop study instruments, categorise data, shape the analysis and guide policy recommendations. For example, in the new vaccine study the framework was modified to be specific to vaccination services, with sub-categories identified in each building block (e.g. within ‘human resources’ were availability of staff, training, remuneration, satisfaction and supervision). The questionnaires were structured according to the building blocks (see Table 2 for examples).

**Table 2:** Examples of questions structured according to the building blocks

**Building block**	**Measles study**	**New vaccine study**	**Polio study**
**Service delivery**	Do measles campaigns affect your capacity to reach remote areas for routine outreach services?	Has the number of outreach activities changed because you started offering the new vaccine?	Are routine immunization activities affected during polio campaign days?
**Health workforce**	Do measles campaigns take staff away from routine activities?	Did the training focus solely on the new vaccine or did it cover issues relevant for other vaccines or health services too?	Are health workers’ motivation levels the same as before polio campaigns began?
**Health information system**	Was there any change in the processes for identifying high risk groups and their vaccination coverage rates?	Have immunisation documents been reprinted to include the new vaccine? If yes, has this changed the time required and data completeness?	Has the surveillance system changed as a result of polio?
**Medical products, vaccines and technologies**	Have measles campaigns lead to additional infrastructure, such as waste management equipment?	Has the cold chain capacity related to the new vaccine had any impact for products other than vaccines, such as ARVs?	Have there been any changes in the cold chain infrastructure over the last 10–15 years? Are any of these changes a result of polio?
**Financing and sustainability**	Have donor funds been earmarked to measles campaigns?	Has funding requirements for the new vaccine affected the level of funding for other routine health related activities?	Is funding for polio separate from other health programs?
**Leadership/governance**	Do you think measles campaigns tend to strengthen or weaken policy processes?	Did the planning for the new vaccine have any effect on planning activities of other health services?	In the past, have government officials given a high level of attention to routine immunization activities? Has this changed as a result of polio?

In the three studies, thematic content analysis was used to explore the interview data using the modified Building Blocks framework to map and chart data. As described in Table 2, this analysis was triangulated with data obtained through staff observation, document review, and staff facility surveys.

## Critical reflection on the use of the Building Blocks framework for assessing the health systems impact of disease control programmes

### Advantages

The Building Blocks framework proved useful in several ways. We found that it presented a simple manner to discuss key functions of the health systems in fora that involved a number of researchers from different backgrounds. The ubiquity of the framework provides a common language and facilitates a shared understanding that is useful; one common framework in the international community is more helpful than having multiple potential frameworks. Its common use made it an easy choice for us to select for our studies – no other health systems framework is as well used. Hence, as researchers, we did not need to ‘reinvent the wheel’ by creating a new framework. The Building Blocks framework helped to structure research questions and data collection tools, ensuring that all important health systems functions were covered. Finally, having such a common framework might allow for easier comparison between studies.

### Limitations

#### Translating a framework into an analytical tool

The Building Blocks framework was not originally intended to be an evaluative instrument, but was developed for guiding the investment of resources in health systems [18]. As a result, it is organised around a supply model that features detailed aspects of service delivery, but remains mostly silent on demand side aspects. It also does not include social mobilisation activities, which can be a critical component of health systems in low- and middle-income countries. This gap forced our three studies to incorporate demand-side aspects (e.g. community demand and social mobilisation activities) somewhat artificially into the service delivery component.

The six Building Blocks are considered as a set of inputs that contribute to the desired outcomes of a health system, improved health and health equity, responsiveness, social and financial risk protection and improved efficiency, through improving access, coverage, quality and safety [19]. However, this view, taken up in both the WHO literature and among researchers [14,19-23], easily leads to neglecting the links between these three main components (inputs, outputs and outcomes).

Along the same lines, as others have noted, the literature is relatively silent on the interactions between the six building blocks in the framework [18] and, as a result, it remains rather static [24,25]. For example, in the polio study, information was gathered regarding the financing of polio, routine immunization and other programs. The final report contained a discussion of financing that integrated information from all eight case studies. While this allowed a quite nuanced understanding of the variations in financing mechanisms for polio around the globe and the concrete effects of various approaches on financing for other health programs, it was not particularly good at tying those trends to factors in other building blocks—for example leadership and governance—despite the fact that governance factors are usually very important in determining how money is used. This approach also failed to bring attention to larger, complex issues: for example, the impacts of large amounts of earmarked money from abroad on the abilities of communities to set their own health agendas.

The framework offers no weighting between the blocks. Hence, all parts of the system are assumed to have the same importance, which is rarely the case in practice. For example, there might be compelling reasons to believe that for vaccination campaigns, the health workforce has a greater impact on health outcomes and the overall system than, say, health information systems. However, when using this method there is no obvious method to reflect this. Others have argued that leadership and governance, the health workforce and the community - make up the core of health systems [24].

#### Using the building blocks framework in applied research

By its very nature, the framework presents challenges in conducting applied research and interpreting results. Questions about the effects of a disease control programme on individual building blocks may unwittingly increase the risk of bias. It could be argued that asking about impacts on specific building blocks constitutes a leading question, suggesting that the researchers expect to see an impact and so increases the risk of acquiescence bias. For instance, by asking specifically about surveillance or the cold chain, respondents may feel inclined to report an effect rather than allowing them to report only the effects that they themselves had noted without prompting. The framework may also encourage researchers to include all building blocks in their study, even though some blocks or dimensions may be irrelevant for the topic under study. For example, in the new vaccine study, questions were included on vaccine wastage, despite there being few reasons for its inclusion beyond being part of a building block category.

In all three studies, it was difficult to gauge perceptions of impact beyond an interviewee’s area of specialty. The segregation of staff by activities and the often narrow focus of programme staff proved challenging when trying to explore broader health impacts. In practice, Expanded Programme on Immunization (EPI) staff often only commented on EPI activities; logisticians could discuss cold chain and procurement issues, but not staffing or other issues, whilst non-EPI staff often knew little about EPI interventions. This tended to be less true at the very top or the very bottom of the governance hierarchical pyramid. Overall, we found that very few interviewees had a bird’s eye view of various health systems components and interactions between programmes and could comment competently on issues, such as distortion of priorities and opportunity costs.

One way to circumvent these problems is to modify the framework for the specific needs of each research study. Formative research should identify which categories are most pertinent to the research question, in order to focus the study more precisely. In the polio study, an extensive literature review was used to identify specific arenas in which polio eradication activities might be expected to show an impact (either positive or negative). This led to the inclusion of some new categories and the minimising of others. For example, the study systematically evaluated whether community members were dissatisfied with a perceived focus on a single disease — a dynamic that the building blocks would not easily capture. Similarly, because polio eradication was not expected to affect medical products beyond the cold chain, interview questions focused on the cold chain alone, and did not ask questions about other issues, such as the vaccine supply chain [26].

When analysing our data, we found that the Building Blocks framework tended to lead to a mechanical and descriptive analysis and presentation of results, almost resembling ticking off a shopping list. In most studies using the framework, results are reported according to each Building Block, one by one. However, such a summary can easily become dull and repetitive and it is not possible for the reader to determine the relative importance of each Building Block impact. Such an approach does not inspire the development of overarching messages for policy makers.

#### Assessment of contributions to health systems strengthening

Guidance on how to assess the effects of health system strengthening efforts on each of the blocks is limited [27,28], and the lack of nuance in the framework regarding how they are structured and linked to each other can make analysis challenging. The framework, indeed, does not easily lead to understanding of why effects were amplified or diminished through linkages with other blocks and this can make it difficult to discern an overall conclusion beyond a list of positive and negative effects. For example, in many regions covered by the polio study, it was clear that the surveillance system had been strengthened, but only for detecting polio. Does the framework in such a case imply that by default the whole system is strengthened? Or could an intervention that is positive for the polio programme at the same time be negative for the rest of the system? This might be where differences between supporting specific components of the system and strengthening it in a holistic and systemic manner might come into play, as highlighted by Chee and colleagues [29].

Another problem is how to capture the time dimension. How could short term gains that may have unintended consequences for the system in the future be identified and mapped? For instance, in the measles study, we found that additional resources were made available for surveillance in Cameroon from polio funds, but with no medium term sustainability plan. The creation of a parallel surveillance system alongside national systems posed questions about its sustainability, which the Building Blocks framework was unable to help resolve. The durability of health systems over time, or the sustainability of strengthening activities, is not captured within the framework.

Given the lack of clarity of what strengthening actually means in this framework, there are serious questions about the ability of the Building Blocks approach to accurately capture health system strengthening effects and opportunity costs. Because they contribute in certain ways to nearly all the individual building blocks, single-disease vaccination campaigns, for example, appear to strengthen health systems according to this framework (as the polio surveillance example above illustrates). However, the framework is not configured to assess whether the funds could have been better spent elsewhere to strengthen the system in a more holistic and sustainable way. This is especially relevant for vaccination campaigns; funding concurrent campaigns detracts funds from activities that could strengthen the broader health system, but this aspect cannot be analysed when using the Building Blocks framework. This point remains generally valid for all disease-specific control programmes. The problem relates back to the point made earlier regarding the lack of an overarching health system perspective within the framework, as well as the neglect of the interactions between the Building Blocks.

## Discussion

Assessing the impact of a programmatic intervention on health systems can be a daunting task because of the absence of any controls or counterfactuals, and the complex, dynamic nature of health systems. On reflection, we found that many weaknesses of the Building Blocks framework resulted from its very elegance and simplicity. A framework is, indeed, needed to support both researchers and policy makers in explaining the reality of health systems, and to help simplify what is complex in order to facilitate policy making. Therefore, despite its many limitations, the WHO Building Blocks framework is valuable as it provides a common language and reference for researchers and policy makers. This is arguably of more use than the creation and use of different frameworks for each study conducted or policy issue raised. Nonetheless, the fact that it is a common framework means there are also common blind spots.

The simplicity allows specific effects of an intervention on individual building blocks to be described adequately. However, this comes at a cost. First, it does not allow to capture the dynamic interactions between the elements of a health system, a key feature that makes health systems complex. Second, the Building Blocks model does not provide any rationale of what makes health systems tick. As a result, the overall effect on the health system of a specific intervention might still be poorly understood and possibly even misinterpreted.

The Building Blocks framework considers health systems to be *complicated*, suggesting that one can describe the system by detailing all of the building blocks within it (“the sum of the building blocks is the whole system”). However, health systems are *complex;* like a living organism, they are dynamic, with interacting components - at various geographical levels - that lead to adaptation and to the emergence of new dynamics. These interactions can be both predictable and unpredictable. They generate feedback loops that will continue shaping the systems and its different components. The WHO Building Blocks framework does not capture this complexity and, consequently, is not well suited to research on the interaction of programmes with health systems.

To avoid falling into the trap of conceptualising health systems as a black box [30], a health systems framework needs to make its assumptions concerning the role and relative importance of the components explicit, and especially how they interact. Indeed, the main weakness of the Building Blocks framework comes from the assumption that scrupulous description of specific effects on all the individual building blocks helps to understand the system as a whole. In reality, it simply presents a checklist of six functions. This is reinforced by most of the guidance on measuring health system strengthening that uses the Building Blocks framework, which just provide generic indicators for sub-dimensions [31]. The framework, indeed, neglects a ‘whole system’ perspective.

We also found that demand-side issues were missing from the framework; others have also noted this [18] and other missing elements, such as behaviour change [32]. However, our criticism is not simply of the framework, but also of how it is used, particularly the focus on the six building blocks to the neglect of the process and outcome aspects of the framework.

The overuse of the Building Block framework poses a risk of considerable “group think” and may contribute to a lack of critical appraisal of health systems and a persistent view that health systems can be fixed as if they were complicated instead of complex (i.e. by only addressing individual components in silos, rather than considering the system as a whole).

There are a number of options for progressing the use of health systems frameworks in research. One option is to ensure that the existing WHO framework is used to its maximum potential. We believe that if the Building Blocks framework is to be used in research, it should be used in a flexible manner, including process and outcome components rather than focusing solely on the six key functions. Another way forward might be to modify the framework before using it for a new research study. Formative research could develop an explicit hypothesis of how the intervention under study may affect the health system, leading to a modified model, which then can be rigorously piloted. Issues of demand, power, process, decision-making and accountability should be explicitly considered in such a modified model. This would have the benefit of better focusing the study on issues pertinent to the topic under study whilst retaining the benefits of a common framework. A more radical option would be to identify an alternative framework that is better suited to research exploring the impact of programmes on health systems. One possible framework is that developed in the World Health Report 2000, which was used to assess the relationship between key functions and objectives of a health system [33] in an attempt to evaluate change to the systems as a whole rather than to the sum of its parts. Alternatively, there may be scope for revising the Building Blocks framework, for example as suggested by Savigny & Adam [31]. They acknowledge that it lacks interactions between components, arguing pointedly that it does not constitute a health system. They attempt to introduce dynamic thinking into the framework, but do not provide a clear way forward on how to operationalize this in a research project.

## Summary

As with frameworks in general, the WHO Building Blocks framework is valuable because it creates a common language and shared understanding. Its simplicity and universality are its strengths. No framework is ideal, but a framework is only as good as the understanding it can generate. For applied research, the Building Blocks framework falls short of what is needed to holistically evaluate the impact of specific interventions on health systems. We believe it would be improved by making four amendments: integrating the missing “demand” component; incorporating an overarching, holistic health systems viewpoint; explicitly including considerations of decision-making and power; and including scope for interactions between components.

Nevertheless, the WHO Building Blocks framework should not be used automatically nor without careful consideration. We believe that if this framework is used, it should be in a more flexible manner than we and other researchers have done to date. It should be adapted according to the specific research question through formative research and piloting. It should be focused on systemic research hypotheses rather than exploring each and every possible effect on sub-components of the building blocks.

Continued critical reflection of and debate around its role and potential for development is necessary if the field of health systems research is to keep progressing.

### Ethics

The manuscript is submitted in the “debate” section and therefore is not a research paper. However, the three studies that are discussed in the papers have all received ethical approval from their implementing institutions (the London School of Hygiene and Tropical Medicine (2009 and 2010) and Middlebury College (2011). Ethical approval was gained from all the countries where fieldwork was conducted, unless the country did not require an ethical approval.

#### Research projects discussed in the debate paper

Project: New Vaccine, from licensing to adoption: ethical approval from the LSHTM number 5739.

Project: Impact of measles eradication on health systems: ethical approval from the LSHTM number 5596.

Project: Impact of polio campaigns on health systems: Middlebury College, ethical approval number 11196.

## Competing interests

We declare that we have no conflicts of interest in the authorship or publication of this contribution (financial or non financial).

## Authors’ contributions

All authors have contributed substantially to the conception, design, writing/drafting and critical revising of the publication. All authors have given final approval of the version to be published and have agreed to be accountable for all aspects of the work in ensuring that questions related to the accuracy or integrity of any part of the work are appropriately investigated and resolved. SMJ has led the drafting of the paper and UKG, SC, HB, and BM have all substantially contributed to various drafts of the manuscript and reviewed the final version of the paper.

## Pre-publication history

The pre-publication history for this paper can be accessed here:

http://www.biomedcentral.com/1471-2458/14/278/prepub
